# Establishing a trusting nurse-immigrant mother relationship in the neonatal unit

**DOI:** 10.1177/09697330211003258

**Published:** 2021-07-20

**Authors:** Nina Margrethe Kynø, Ingrid Hanssen

**Affiliations:** Lovisenberg Diaconal University College, Norway; Oslo University Hospital, Norway; Lovisenberg Diaconal University College, Norway

**Keywords:** Clinical ethics, empirical approaches, ethics and children in care, existentialist, neonatal care, qualitative research

## Abstract

**Background:**

In the neonatal intensive care unit, immigrant parents may experience even greater anxiety than other parents, particularly if they and the nurses do not share a common language.

**Aim:**

To explore the complex issues of trust and the nurse–mother relationship in neonatal intensive care units when they do not share a common language.

**Design and methods:**

This study has a qualitative design. Individual semi-structured in-depth interviews and two focus group interviews were conducted with eight immigrant mothers and eight neonatal intensive care unit nurses, respectively. Data analysis was based on Braun and Clarke’s thematic analytic method.

**Ethical considerations:**

Approval was obtained from the hospital’s Scientific Committee and the Data Protection Officer. Interviewees were informed in their native language about confidentiality and they signed an informed consent form.

**Results:**

Trust was a focus for mothers and nurses alike. The mothers held that they were satisfied that their infants received the very best care. They seemed to find the nurses’ care and compassion unexpected and said they felt empowered by learning how to care for their infant. The nurses discussed the mother’s vulnerability, dependency on their actions, attitudes and behaviour.

**Discussion:**

Lack of a common language created a challenge. Both parties depended on non-verbal communication and eye contact. The nurses found that being compassionate, competent and knowledgeable were important trust-building factors. The mothers were relieved to find that they were welcome, could feel safe and their infants were well cared for.

**Conclusion:**

The parents of an infant admitted to the neonatal intensive care unit have no choice but to trust the treatment and care their infant receives. Maternal vulnerability challenges the nurse’s awareness of the asymmetric distribution of power and ability to establish a trusting relationship with the mother. This is particularly important when mother and nurse do not share a verbal language. The nurses worked purposefully to gain trust.

## Introduction

For parents it is a worrisome and stressful experience when their newborn infant needs to be admitted to a neonatal intensive care unit (NICU).^1^ They have no choice but to leave the fate of their newborn in the hands of the professionals and trust that they will give their infants the best treatment and care possible. This is a situation that makes all parents vulnerable and dependent^2^ as the knowledge difference between themselves and the healthcare professionals renders it difficult for them to challenge the professionals’ judgement.

Primo March 2020, 14.7% of the Norwegian population were immigrants.^3^ When the parents are immigrants, they may meet an unknown language, an unknown structure and an organisation of the healthcare system that is difficult to comprehend, as well as a new and unknown societal culture.^4^ This may lead to even greater anxiety and insecurity than in other parents and make it more difficult to participate actively in the care of the child. This may in its turn challenge their attachment to the child and lead to increased stress and worry for the parents.^5^

## Background

There is no agreed-upon definition of the concept of trust.^2^ However, all patient–healthcare professional – in our case parent–nurse – relationships are more or less asymmetric. ‘This epistemic asymmetry is caused by one party having skills and knowledge that the other party does not have but needs’.^6^ This asymmetry becomes even greater when the parents are immigrants who do not have a common language with the nurses and cannot communicate verbally without an interpreter present.^7^ The maternal vulnerability challenges the nurse’s awareness of the asymmetric distribution of power. To establish a trusting relationship with the mother, nurses need to be attentive towards the mother’s needs.^8^

In the NICU, the mother is usually the primary parent during the hospital stay. Building trust is a challenge in a setting where the mothers are anxious about the fate of their newborn infant. Parents tend gradually to become a more active part in the daily care of their baby as they become more comfortable with the unit’s setting and their infant’s situation.^9^ The authors describe this as a partnership between parents and nurses that develops in three phases: the acute critical phase, the stabilising phase and the discharge phase. The first phase seems to be the most challenging as this is the primary period in the development for the parent–nurse relationship. In this phase trust needs to be fostered while the infant’s situation is at its most precarious. One might presume that becoming comfortable with the unit’s setting and their infant’s situation is even more challenging for immigrant mothers than for mothers who can converse with the healthcarers, ask questions and be continuously informed.

Collaboration with the mothers is very important. The nurses need to be trusted to do their work skilfully and professionally. This is supported by Grimen^10^ who points out that there is an important collaborative and transactional aspect in a trusting relationship between healthcare providers and patients. According to the Danish philosopher Løgstrup,^11^ trust is a silent ethical demand and part of the human existence. If our trust has not been damaged by previous experiences, we meet each other with mutual trust. As the mothers have no option but to trust their healthcare providers, the ethical demand on the nurses to care for infant and mother becomes even more profound as a person through trusting places ‘something of his own life into the hands of the other person’.^11^ Løgstrup^11^ furthermore points out that ‘[a] person never has something to do with another person without also having some degree of control over him or her’. In such a context Dinç and Gastmans^12^ hold that trust has a normative meaning, because nurses must direct their focus and care towards meeting needs and resolving problems. Trust is a humane prerequisite.^13^

The mothers have no option but to trust their healthcare providers, a situation that makes trust include dependency.^6^ Thus, parents have no other choice than to leave the care of their fragile infant into the hands of the healthcare personnel. This is an expression of the ethical demand and uncovers the nurses’ responsibility in care of the infant and parents.^14^ This shows that nursing is to be understood as a relationship-based practice. Since care is the basis of nursing practice, it is based on (1) moral practice, (2) relations and (3) practical care.^13,15^ Mothers in the NICU trust competent nurses who act friendly and skilfully, though former experiences influence their trust.^16^ Feeling safe is incorporated in this trust. We argue that a person cannot trust unless he or she feels safe.

The aim of this article is to explore the complex issues of trust and the nurse–mother relationship in NICUs when they do not share a common language. The research questions are as follows:

- How do nurses work to create trust in mothers with whom they share no verbal language?
- What makes mothers experience trust in the nurses?

## Methods

The study has a qualitative design, triple methods approach with individual semi-structured in-depth interviews of mothers and focus group interviews of nurses. Participant observations were also conducted. In this article, we present data from the interviews only. The purpose of the interviews was to learn about the interviewees’ experiences, attitudes, thoughts and motives through letting them speak freely and in their own tempo.^17^

*Participants*: Eight mothers and eight nurses were interviewed. The mothers hailed from Europe, Asia and Africa and had from none to two children before their present hospitalised infant. Their stay on the ward varied from a few days to 2 weeks. The nurses’ NICU work experience varied between 2 and 34 (mean 16) years. Five of them were nurse specialists with post-graduate education in intensive care, paediatric or neonatal nursing.

*Inclusion criteria:* Mothers aged more than 18 years, who neither spoke any Scandinavian languages nor English. Their infants had to be physiologically stable at the time of the interview as evaluated by the unit nurses. Recruitment and interviews took place towards the end of the infant’s hospital stay. The immigrant mothers who were invited to participate as interviewees in the study were suggested by unit nurses based on their assessment that an interview would not be too much of a burden. Oral information about the study was given through interpreters. Nurses working in the NICU who volunteered to participate in a focus group interview were also included.

*Exclusion criteria*: Mothers whose infants were in an acute or critical phase, or who were younger than 18 years old. Mothers and nurses who were able to communicate verbally with each other were also excluded.

### Data collection

A total of 16 participants were interviewed. First, the mothers were interviewed assisted by professional interpreters as seven different languages were spoken. The nurses’ experiences were explored through two focus group interviews with four nurses in each group.

During the interviews, a semi-structured interview guide was used. The questions were open-ended to encourage the mothers to talk freely about their experiences in the NICU. The main questions were concerning whether they felt that they received all the information they needed about their infant’s illness, treatment and care, and the daily routines in the NICU. Moreover, we asked whether being unable to understand what the nurses said could make them feel suspicious, and whether they were satisfied with how communication and interaction with the nurses were facilitated. These interviews lasted 25–35 min and took place in a quiet room in the ward.

To conduct interviews through interpreters is more demanding than same-language interviews as the researcher is totally dependent on the interpreters’ ability to communicate not only the lexical words but also the meaning of what is being said. It was therefore important that all the interpreters were well qualified and experienced.

Each focus group interview with nurses lasted about 40 min and produced rich data through narratives, complementary comments, questions and answers. Field notes were taken during the entire data collecting period.

### Data analyses

The interviews were audio recorded, transcribed verbatim and checked for accuracy against the recordings. The first author is a neonatal nurse specialist, while the second author is not and therefore had an outsider view on the data. Thus, we focused on minimising bias and strengthening trustworthiness. Both authors took part in the data analysis which was thematic and hermeneutic in character where depth of understanding was attained through a circular investigation of the interviews.^18^ Rigour was obtained through following Braun and Clarke’s^19^ phases of thematic analysis: In phase 1, we familiarised ourselves with the data. In phase 2, interesting features were coded and collated into potential themes (phase 3, searching for themes). These codes were developed through discussions between the researchers. Together we identified patterns across the data set in relation to the research question. Phases 4 (reviewing themes) and 5 (defining and naming themes)^19^ were done collaboratively, while in phase 6 the first author wrote a preliminary paper text which we then discussed and developed further collaboratively. During the thematic analysis we read and re-read the interview texts and thus strived to ‘remain open to the meaning of the other person or the text’.^18^ This created a circular investigation of the transcribed interview data where each reading led to greater depth of understanding of the data corpus.

Quotations/telling meaning units are presented to emphasis our findings.^20^ This also strengthens the study’s confirmability as it shows that the findings are based on our interviewees’ responses and not on potential bias or any personal motivations that would skew our interpretations.^20^ Dependability is achieved through presenting the basic questions asked during the interviews and following the chosen model for data analysis step by step. Thus, it will be possible to repeat the study in a similar setting by other researchers. And finally, trustworthiness through transferability is achieved by presenting thick descriptions to show that the study’s findings can be applicable in similar contexts, circumstances and situations.

### Ethical considerations

The study was approved by the Oslo University Hospital’s Scientific Committee and the hospital’s Data Protection Officer (certificate number 17/16915). All potential interviewees received written information, and orally information through interpreters, in their home language. They were informed about confidentiality, that participation was voluntary and that they could withdraw from the project at any time. All interviewees signed an informed consent form. Recorded interviews were deleted after transcription.

## Results

Data showed that interpreters were rarely used in the daily nurse–mother interactions. The interviewed mothers spoke spontaneously about trust and how they felt safe during their hospital stay. This was reflected by the nurses who in the focus group interviews discussed the importance of trust and how they worked to achieve a trusting relationship with the immigrant mothers.

### Turning uncertainty into trust

Mothers of newborns admitted to the NICU are vulnerable in many respects. This is not the least the case in immigrant mothers who do not have a common language with the nurses: ‘I feel that they are particularly vulnerable, of course. They come here to our department which is very, very different from what they are used to maybe?’ (Nurse F). This was discussed a lot during the focus group interviews.

The nurses could not know whether the mothers had any confidence in the healthcare system in general or nurses in particular when they came to the ward. Moreover, with no common verbal language, the nurses were unable to ask the mothers about previous experiences. Therefore, the nurses wondered whether the NICU environment seemed strange and frightening to the mothers: ‘We do not know what background they have,…Even the monitor may seem scary; all [the equipment] around them. We know very little about [how they feel about] that’ (Nurse S).

Promoting trust in their relationship with the mothers was something the nurses discussed a lot. While unable to ascertain whether the mothers had basic trust or not, they all agreed that ‘we need to make sure that the mother feels safe’ (Nurse A). Making the mothers feel safe was seen as the ‘starting point’ for all nursing actions. Providing competent nursing was an important way to achieve this: ‘I try to show her that I am able to take good care of her baby’ (Nurse S).

The mothers indicated that they did not necessarily come to the NICU with a basic feeling of trust. Some mothers told about their surprise at not having been chased out of the NICU: ‘They don´t chase me out [when on the ward]. It’s not just me, they are not chasing anyone else out, either’ (Mother D). However, the mothers were very much aware of their dependency: ‘My baby is in the hands of doctors and nurses and I think that the baby is in safe hands’ (Mother U), and most importantly, ‘They take good care of my baby’ (Mother B).

The importance of the mothers feeling welcome and being included in the care at the NICU were much discussed by the nurses. Nurse A held that ‘one of the most important things…must be that they feel welcome’ in an environment they suspected was rather alien to the mothers. This nurse found that her facial expression was important to come across as welcoming and to show that ‘I am glad you have come here to us’ (Nurse A). As a reflection of this, the mothers said that they experienced the NICU as a welcoming environment. They could stay on the ward together with their infant and were included in care of their baby. A mother related that this ‘feels very good. You are relieved when you are well received, when they look at you in a pleasant way and say hello…I have enough time with my child, I can stay as long as I want’ (Mother W).

Mothers held that they were satisfied that their infants received the very best care because the infants seemed to thrive. Thus, the mothers developed trust in the nurses through learning to trust their competency. Even when the mothers did not understand what the nurses did, they trusted them: ‘I was not afraid. I had confidence in what they were going to do.…And then of course I wanted to understand it’ (Mother B). The latter point, to help the mothers understand what and why the nurses did as they did, was a central aspect in the nurse–mother relationship. To make the mothers’ stay as good as possible and for them to learn how to care for their infant’s, Nurse L said that ‘I try to give them tasks to do’. Nurse E found ‘that the mother understands that [she is being] included and she understands that she is welcome to participate’. This the nurses thought increased the mothers’ experience as a carer and made them feel in control. The mothers seemed to agree with this as they expressed that they felt empowered as they were given the opportunity to learn how to care for their infant. Mother I said that ‘I decide, [but] when it comes to milk and stuff, it’s really up to them. Because they are those who know this better’. When directly asked if they felt safe, the mothers said that they did feel safe: ‘I must say that both doctors and nurses – the healthcare personnel – take very good care of you!’ (Mother G).

### Nursing compassion

As the nurses tended not to know the background of the immigrant mothers on arrival, this uncertainty influenced the way the nurses approached them. They tried to read the mothers’ facial expressions and felt they could gauge their mood throughout the shift. All the nurse interviewees found the mothers often ‘smiling with joy’, and they held they could establish their feelings when they had a ‘calm face’ and said ‘goodbye’ when the nurses left the NICU for the day. And, ‘most people understand what a sad face looks like’ (Nurse F). This way the nurses paid attention to the mothers’ feelings and state of mind the best they could.

The nurses found it essential to have and show compassion. They spoke about behaving motherly and making the mother feel safe by saying things like ‘It´s fine…I´m here to be kind to you and your baby’ (Nurse H). ‘I want to show that *here you are safe*. It becomes one of those mutual good things, even though we cannot speak [the same language]’ (Nurse H).

An important phenomenon discussed among the nurses was the mother’s dependency on their nursing actions, attitudes and behaviour. Nurse E pointed out that ‘you are responsible for this person’s life and have this person’s life in your hands. You feel that you cannot go off for the day until you’ve done something [about the situation], and this does affect you. It is such a great responsibility for a fellow human being’. The experience that the mothers depended on them triggered the nurses, and they worked hard and with compassion to achieve a trusting relationship with the mother. That several nurses often did not leave the NICU at the end of their shift was justified by their feeling that they betrayed the mothers’ trust if they left them before everything was calm and well organised: ‘I felt I couldn’t leave work, because they [the parents] were completely desperate’ (Nurse Z).

In line with this one of the mothers found that the nurses ‘are concerned about me, showing that they care about me and my child. [They] are attentive’ (Mother I). Mother U was impressed with the way the nurses conducted themselves:

They do their tasks with very much *true feeling*!…It is both the way they touch and the way they hold the child, the way they treat the child. You feel safe. You do not feel alienated and the way they do things make that trust grow within us.

Furthermore, the nurses ‘act in a very calm and pleasant way…I can feel it…it’s overwhelming’ (Mother P). The mothers seemed to find the care and compassion demonstrated by the nurses to be unexpected. Some of them compared the nurses’ comportment with the care they previously had received from their family in their home country:

I have been here for a while and have had close contact with them [the nurses] and saw this kindness in these people.…. they have been better than my sisters and brothers if they had been physically here with me. (Mother P)

## Discussion

Attentive conversations and small talk between nurses and mothers tend to lead to a trusting relationship and increase the mothers’ experiences of trust.^21,22^ This is not possible when a common language is missing, and it is challenging to understand and decode the mothers’ non-verbal language.^8^ Without a common language, daily communication and information giving between nurses and mothers admitted to the NICU must be carried out through non-verbal communication, eye contact and guesswork.^7^

### Creating trust as an ethical enterprise

Løgstrup^11^ claims that trust is an anthropological part of human existence. Trust is basic in all human relations and is an unspoken ethical demand, and only through negative experiences does distrust arise. Some of the mothers have probably had previous experiences that had given them reasons to be mistrustful. This seems to be strongly indicated by their surprise and happiness of not being harassed or asked to leave the ward. Their reactions tell a story of initial distrust. The nurses therefore had to work purposefully to gain the mothers’ trust. To mitigate worry and possible distrust, they used their nursing actions to show confidence, competency and care.

If mistrust in the nurse–mother relationship arises, the mothers will need a long time to restore trust.^16^ Therefore, awareness of and reflection upon phenomena such as trust, distribution of power and non-verbal language are important. Thus, the nurses were very much aware of the transactional side of trust between mother–nurse relationship.^10^ The infants’ mothers entrust their most precious possession into the nurses’ custody. This leaves them vulnerable and dependent on the nurses doing their best to meet the newborns’ various needs. The nurses did their best to show that they truly cared through their communication as well as their comportment.

The well-known metaphor ‘holding someone’s life in your hand’ was used by both nurses and mothers. To the nurses this expressed their experience of the immigrant mothers’ vulnerability and dependency and how they met this with responsibility and by taking special care of them. The mothers used the metaphor to describe their own experience of dependency by saying how the baby’s life was in the hands of nurses and doctors. They had trust because their babies thrived and were cared for in a competent and thoughtful manner.

Although former experiences influence whether the mothers trust the nurses,^16^ our data clearly indicate that immigrant mothers in the NICU trust competent nurses who act friendly and skilfully. Feeling safe is an explicit component of experiencing trust. The nurses seemed to realise that ‘trust cannot survive, let alone flourish, in an environment of distrust’.^23^ Because they were not sure if the mother came in with trust or distrust, they used different nursing strategies to initiate trust. The lack of a shared language constituted a great challenge and reveals the asymmetric power distributed and the mothers’ dependence on the nurses. Within this dependency lies an inherent demand that nurses must be conscious of this asymmetric power and the mothers’ vulnerability. This the nurses seemed to be very much aware of as they did their uttermost to create a safe atmosphere and a trusting nurse–mother relationship. Thus, they conducted their neonatal nursing as a moral practice.^13,15^

### Trust through competency and compassion

The nurses being competent and knowledgeable was an important factor in building trust. It had, however, to be coupled with showing genuine care for the infants and their mothers. The nurses found it essential to have and show compassion. Compassion is an interpersonal phenomenon that we find characterised the nurse–immigrant mother relationship to a large degree. Their compassion and conscience sometimes made the nurses stay on after end of shift to make sure that problems or challenging situations were resolved before leaving.

According to Slettmyr et al.,^24^ nurses are shown to act altruistically with a feeling of unconditional caring responsibility. Our nurse interviewees showed the mothers compassion and were very aware that the mothers needed to feel welcome on the ward and included as participants in the care for their infant. Compassion seemed to be an ethical compass to the nurses to promote nursing as (1) moral practice, (2) relations and (3) practical care.^13,15^ This finding is in contrast to that of Hem and Heggen^25^ who found that nurses are not always guided by compassion in their work. As seen in the ‘Results’ section, the nurses’ compassion was pointed out as an important trust creating factor by the mothers.

The mothers found themselves in a totally dependent situation where leaving their sick infants with the healthcare personnel was their only viable option, whether they trusted them or not. In this vulnerable state, the mothers were relieved to find that they were welcome and could feel safe and well cared for. It is through the nurses’ clinical care for the infant and their various expressions of compassion that the nurses were able to instil a feeling of trust and safety in the mothers. In this clinical setting, mother and nurse meet in a relational and clinically transactional setting that has the potential for two outcomes: trust or distrust. When the result is trust, the mothers feel safe even when they initially do not understand the what and the why of the nurses’ actions.

## Conclusion

The parents of an infant admitted to the NICU have no choice but to trust the treatment and care their infant is given. Maternal vulnerability challenges the nurse’s awareness of the asymmetric distribution of power, and to establish a trusting relationship with the mother, nurses need to be attentive and compassionate towards the mother’s needs. All mothers did not trust the nurses basically, so the nurses worked purposefully to gain trust through competency, compassion and awareness. This is particularly important when mother and nurse do not share any verbal language.

The nurses found that compassion, competence and knowledge were primary factors in building trust. The mothers were relieved to find that they were welcome and could feel safe and their infants were well cared for. This study shows how a trustful relationship can be established on skilful basis.

### Critical comments and limitation

The number of interviewed was adequate to secure information power as all participants held the experiences and characteristics needed to answer the research questions. The interviews uncovered rich and various data. The interpreters were all authorised, highly educated and experienced with hospital settings. Nevertheless, it may be a limitation that the interviews were not back translated to their original languages and checked for accuracy.
